# Automatic positioning of cutting planes for bone tumor resection surgery

**DOI:** 10.1007/s11517-024-03281-y

**Published:** 2025-01-17

**Authors:** Alessio Romanelli, Michaela Servi, Francesco Buonamici, Yary Volpe

**Affiliations:** Department of Industrial Engineering, University of Florence, Via Di Santa Marta 3, 50139 Florence, Italy

**Keywords:** Computer-aided surgery, Tumor resection, Optimization, Surgical planning, Orthopedic surgery

## Abstract

In bone tumor resection surgery, patient-specific cutting guides aid the surgeon in the resection of a precise part of the bone. Despite the use of automation methodologies in surgical guide modeling, to date, the placement of cutting planes is a manual task. This work presents an algorithm for the automatic positioning of cutting planes to reduce healthy bone resected and thus improve post-operative outcomes. The algorithm uses particle swarm optimization to search for the optimal positioning of points defining a cutting surface composed of planes parallel to a surgical approach direction. The quality of a cutting surface is evaluated by an objective function that considers two key variables: the volumes of healthy bone resected and tumor removed. The algorithm was tested on three tumor cases in long bone epiphyses (two tibial, one humeral) with varying plane numbers. Optimal optimization parameters were determined, with varying parameters through iterations providing lower mean and standard deviation of the objective function. Initializing particle swarm optimization with a plausible cutting surface configuration further improved stability and minimized healthy bone resection. Future work is required to reach 3D optimization of the planes positioning, further improving the solution.

**Graphical Abstract:**

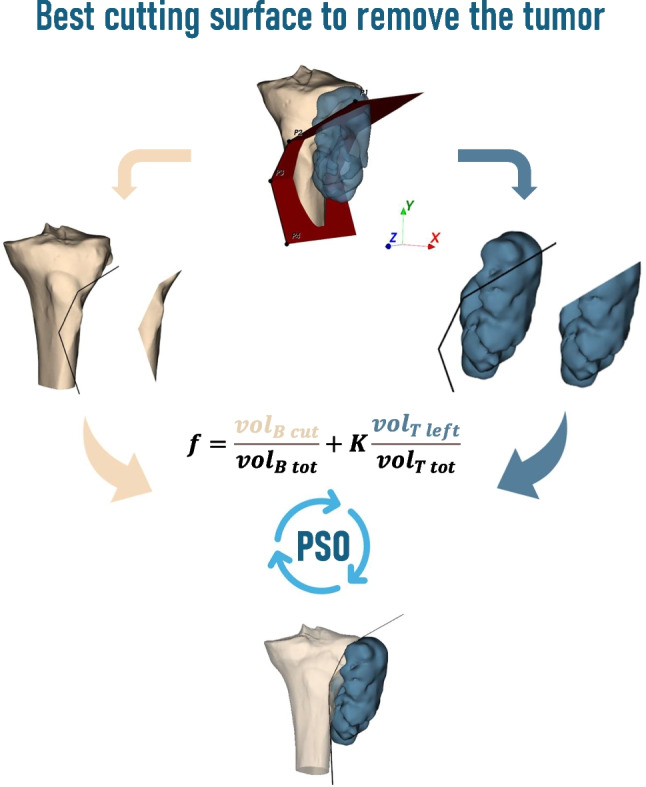

## Introduction

**Fig. 1:**
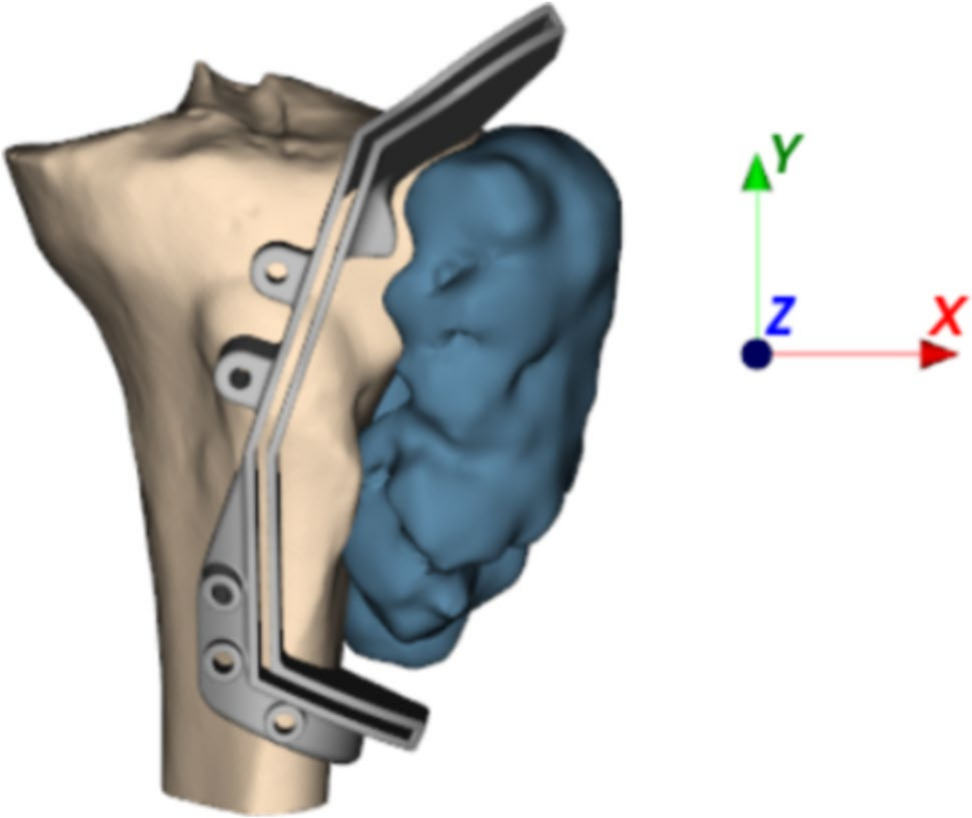
Example of cutting guide (in gray) for bone tumor resection surgery; *z*-axis represents the direction of surgical access

The evolution of Additive Manufacturing and Reverse Engineering has enabled the widespread use of patient-specific cutting guides for bone tumor resection and osteotomies [1, 2]. These guides provide reference surfaces to precisely follow pre-planned bone-cutting paths [3] using an oscillating saw. Designed from the patient’s CT data by engineers following surgeon instructions, the guides are 3D printed using biocompatible materials and currently represent a reliable practice in many hospitals [4–6]. While design details vary in each case, key features include, as visible in Fig. 1, a blade slot for saw guidance along the cutting planes, a base matching bone anatomy for stability, and holes for bone fixation [7].

The modeling process for surgical cutting guides is often complex and time-consuming. To address this, Memon et al. [8] developed a design software specifically for 3D-printed cutting and reconnection guides in cranio-maxillofacial surgery. Other studies [9, 10] devised automatic CAD modeling procedures for the entire guide.

However, the positioning of cutting planes is currently a manual process performed by surgeons based on each patient’s condition and their expertise. This is complex, requiring consideration of tumor location, shape, size, and proximity to vital structures. Automating this process could reduce surgeon workload and minimize the removal of healthy bone, potentially improving postoperative outcomes.

Research has explored automatic cutting plane positioning. Carrillo et al. [11] developed an optimization framework using a genetic algorithm for preoperative planning of corrective osteotomies. Their output includes the position and orientation of the osteotomy plane, fixation plate, and screws.

Zhang et al. [12] implemented an algorithm for positioning multiple cutting planes in bone tumor resections. Their method involved expanding the 3D tumor model per surgeon-defined margins to create a “dangerous region,” then optimizing 2D plane positioning to minimize the area enclosed by planes and the convex hull of the dangerous region projected onto a preferable plane. Finally, 3D positioning minimizes healthy bone volume removed, though initially optimizing an area instead of a volume may lead to local minima.

Hill et al. [13] identified each plane’s position by two rotation angles, geometrically evaluating bone/tumor shape and position relative to potential cutting planes. For each plane, the algorithm rotated 3D bone voxel model and tumor surface model by two angles about the $y$ and $z$ axes, then the potential cutting plane was positioned at the most distant point of the tumor surface in the $x$-axis and perpendicular to the $x$-axis. Optimal positioning was reached using particle swarm optimization (PSO) in a multi-stage process. However, positioning one plane at a time without considering the overall cutting path may fail to minimize healthy bone removal for concave tumors.

This work presents an algorithm that directly generates and optimizes the cutting path defined by multiple planes to accurately follow the tumor surface. A cutting surface is generated by extruding a polygonal chain along an appropriate direction and its positioning is optimized using PSO to minimize an objective function balancing tumor removal and healthy bone preservation. A custom procedure derives an initial plausible cutting surface based on the tumor’s convex hull to improve optimization.

While optimal cutting plane positioning is generally a 3D problem, this study considers some practical constraints that were delineated in collaboration with the surgeons that supervised this work. Primarily, the constraints address the fact that, to minimize the impact on the patient, resections typically entail a single surgical incision to access the area of interest, and therefore access is often limited to a single direction, as can be seen in Fig. 1. Accordingly, the modeling focuses on finding planes parallel to the surgical access direction, reducing it to an almost 2D problem. This simplification greatly reduces numerical complexity while having a limited effect on procedure effectiveness. However, unlike other 2D approaches that minimize an area [12], this work drives optimization towards minimizing the resected volume. Furthermore, the optimized 2D cutting path could initialize a subsequent 3D optimization of the planes positioning.

## Material and methods

The following sections describe the required input data, the algorithm for generating and optimally positioning the cutting surface, necessary controls and penalties in implementation, and an algorithm to generate an initial plausible solution to initialize the optimization.

### Inputs and preliminary operations

To identify the optimal cutting surface, the algorithm requires the following:

- 3D tessellated surface model of the bone, typically obtained from segmented CT images.
- 3D model of the tumor, which can be obtained from the segmentation of MRI images.
- The direction of surgical access indicated by the surgeon.
- Number of planes ($n$) composing the cutting surface. Evidently, a greater number of cutting planes ensures to follow the boundary surface of the tumor more accurately. However, the clinical feasibility must be considered as the width of the saw (generally no less than 20 mm) is a limit to small cuts. The number of cutting planes is thus provided by the surgeon considering also the specific shape of the tumor and the direction of surgical access. Still, this could be varied in a limited range (± 1) to evaluate alternatives.
- A safe margin distance between the tumor and cutting path to ensure complete tumor removal accounting for potential errors.

The tumor 3D model is transformed so that its centroid is at the origin of the reference system with the surgical access direction aligning the $z$-axis, providing the surgeon’s viewpoint during the procedure. This represents the reference system that will be used in the description of the algorithm.

The bone model is roto-translated with the same rotation matrix. An offset equal to the safe margin expands the tumor model to ensure its complete resection when cut.

### Algorithm for the optimal positioning of cutting planes

Given the focus on planes parallel to the surgical access direction ($z$-axis), each cutting surface of $n$ Planar Surfaces (PSs) has an $xy$-plane projection as a polygonal chain of $n$ line segments and $n+1$ points. Therefore, the optimization identifies the optimal $xy$-positions of the $n+1$ points defining the cutting path, corresponding to $2(n+1$) scalar variables.

The algorithm uses particle swarm optimization (PSO), a population-based stochastic method [14]. Each particle represents a potential solution, composed of the $xy$-coordinates of the $n+1$ points initialized randomly within the bone model’s bounding box.

The points must be positioned to minimize the volume of resected healthy bone ${vol}_{B\, cut}$ while ensuring complete tumor removal, i.e., ${vol}_{T\, left}=0$. The objective function balances these goals:

1 $$f=\frac{{vol}_{B\, cut}}{{vol}_{B\, tot}}+K\frac{{vol}_{T\, left}}{{vol}_{T\, tot}}$$

where the volumes are normalized by total bone and tumor volumes, ${vol}_{B\, tot}$ and ${vol}_{T\, tot}$ respectively, and $K$ weights the tumor removal term. Larger $K$ values reduce tolerated unresected tumor, ideally eliminating it for $K\to \infty$. However, this would correspond to a strong constraint that may not be managed by the algorithm since the global minimum of such a function would be very close to points of maximum. Therefore, a weighted objective function integrating both goals is used.

### Objective function

This section explains the strategy for evaluating the objective function terms in Eq. 1. ${vol}_{B\, tot}$ and ${vol}_{T\, tot}$ are computed from the original 3D bone and tumor mesh models using existing volume calculation methods.

**Fig. 2:**
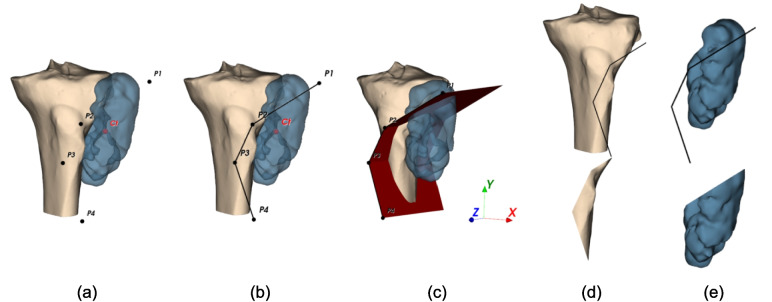
Objective function evaluation procedure: **a** Input points ordered according to polar coordinates calculated with respect to the centroid of the tumor *C*_t_; **b** polygonal chain generation; **c** cutting surface generation by polygonal chain extrusion along the *z*-axis; **d** split operation of the bone mesh using the cutting surface (upper) to obtain resected bone part (lower); and **e** split operation of tumor mesh using the cutting surface (upper) to obtain resected tumor part (lower)

${vol}_{B\, cut}$ and ${vol}_{T\, left}$ depend on the optimization variables, requiring the following steps (Fig. 2):

- Input the $\left(\overline{x },\overline{y }\right)$ coordinates of the cutting path points to evaluate.
- Order the points by their polar coordinates from the tumor centroid (Fig. 2a).
- Generate a polygonal chain on the $xy$-plane connecting the ordered points (Fig. 2b).
- Extrude the chain along $z$ to create the cutting surface (Fig. 2c).
- Split the bone model with the cutting surface to obtain the resected bone part (Fig. 2d bottom).
- Compute $${vol}_{B cut}$$.
- Split the tumor model with the cutting surface to obtain the resected tumor part (Fig. 2e bottom).
- Compute the resected tumor volume and subtract from ${vol}_{T\, tot}$ to obtain ${vol}_{T\, left}$.

This allows the evaluation of the objective function value $f$ for the given points.

While the overall procedure to evaluate the objective function may seem straightforward, several aspects and potential complications need to be addressed during implementation. Therefore, although the major steps were outlined previously, additional operations were introduced to perform checkpoints and apply penalties to avoid invalid cutting path configurations. These sub-steps, described in the following section, ensure the algorithm robustly handles edge cases and constraints.

### Additional steps performed to compute the objective function

The procedure described is implemented in Python language. Different libraries for processing 3D objects were tested leading to the choice of using The Visualization Toolkit (VTK) [15] since it supports a wide variety of visualization algorithms and advanced modeling techniques. How the controls and penalties, that will now be described, were handled greatly depended on the choice of this library.

#### Control on surfaces normals

Since each generated cutting surface comprises multiple planar surfaces (PSs) with different normals, additional checks are required when performing splitting operations between surfaces. The normals must have the correct direction to properly isolate the resected bone and tumor parts.

**Fig. 3:**
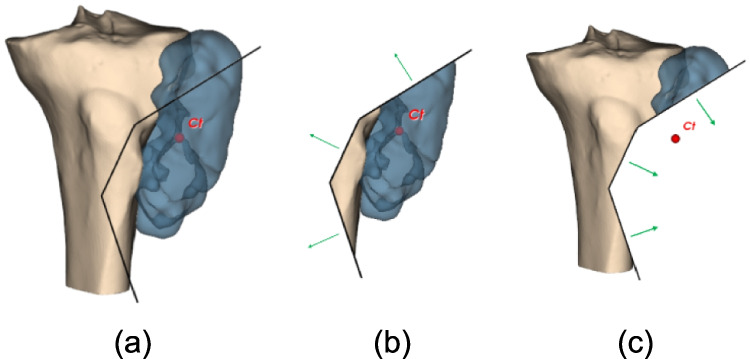
**a** Bone and tumor models with cutting surface; in red the centroid of the tumor and **b** tumor resected part, containing the centroid, and correspondent bone resected part

The resected tumor part is defined as containing the tumor centroid. Consequently, the resected bone part is the bone section removed along with the resected tumor portion (Fig. 3a, b).

For the normals to have the right direction using VTK splitting, they must all point away from the tumor centroid, and inverting the normals would incorrectly compute the parts in Fig. 3c.

However, for more complex cutting surface configurations, the correct normal direction depends on each point’s position relative to the others and the tumor centroid. Three different situations are managed to ensure the normals point in the proper direction.

**Fig. 4:**
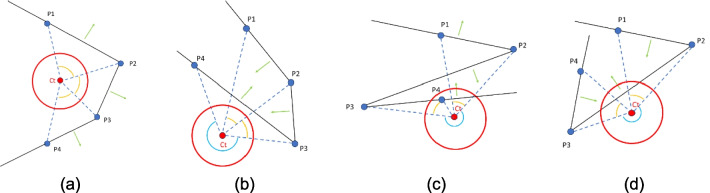
Cutting surfaces (in black) and their normals (in green) with correct direction; in yellow angles between consecutive point-barycenter segments; in blue the angle larger than *π*; **a** case with all angles smaller than *π*; **b** case with one angle larger than π relative to a pair of points forming one of the ending PSs; and **c**, **d** cases with one angle larger than π relative to a pair of points forming an intermediate PS: **c** consecutive point *P*_4_ inside triangle *P*_2_-*P*_3_-*C*_t_; **d** consecutive point outside the triangle

1. For each consecutive pair of points, the segments connecting them to the tumor centroid subtend an angle less than $\pi$ (Fig. 4a). In this case, the normals of the PSs must point away from the centroid.
2. There exists a pair of consecutive points where the segments connecting them to the tumor centroid subtend an angle greater than $\pi$, and this pair generates one of the two ending PSs (Fig. 4b). Here, only the normal of this PS needs to point away from the centroid.
3. There exists a pair of consecutive points where the segments connecting them to the centroid subtend an angle greater than $\pi$, and this pair forms an intermediate PS (Fig. 4c, d). If the next point lies within the triangle formed by this pair and the centroid, the normal must face the centroid (Fig. 4c). If it lies outside, the normal must be opposite to the centroid (Fig. 4d).

#### Control on number of planes performing the cut

**Fig. 5:**
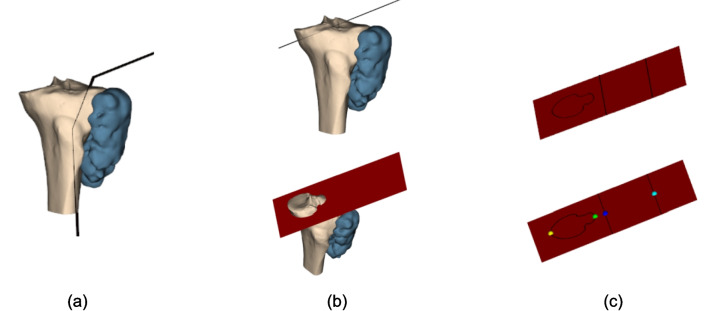
Identification procedure for PS not intersecting the bone mesh: **a** cutting surface with one PS not intersecting the bone mesh; **b** PS corresponding infinite plane; and **c** bone-plane intersection contour outside PS indicated by the two black lines: in yellow contour point with minimum *x*, in green contour point with maximum *x*, in blue and light blue respectively the point with minimum and maximum *x*, between the two that generates the PS

Problematic configurations where one of the PSs does not intersect the bone can occur during the particle swarm optimization (PSO) process (Fig. 5a). To address this, a procedure identifies such cases for penalization:

1. Construct the infinite plane for each PS (Fig. 5b).
2. Obtain the bone-plane intersection contour (Fig. 5c).
3. Check if the contour lies on the PS by comparing the max/min $x$-coordinates of the contour (green, yellow) with the max/min $x$-coordinates of the PS endpoints (blue, light blue). If the contour $x$-range lies outside the PS $x$-range, the PS does not intersect the bone (Fig. 5c).

Configurations in which one of the PSs did not intersect the bone were penalized by assigning an arbitrarily high value to the objective function.

#### Handling of self-intersecting surfaces

**Fig. 6:**
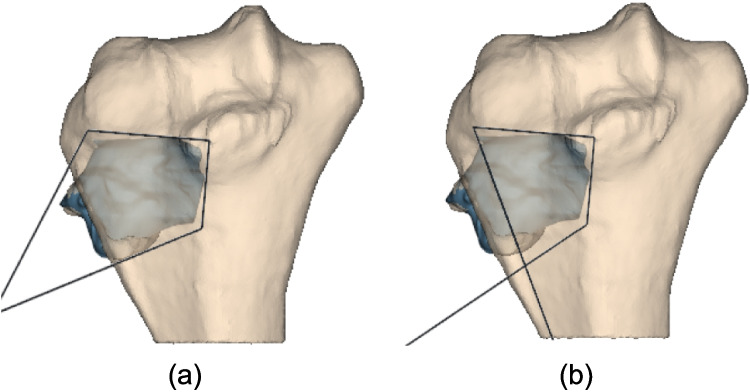
**a** Cutting surface self-intersecting outside bone surface and **b** cutting surface self-intersecting inside bone surface

**Fig. 7:**
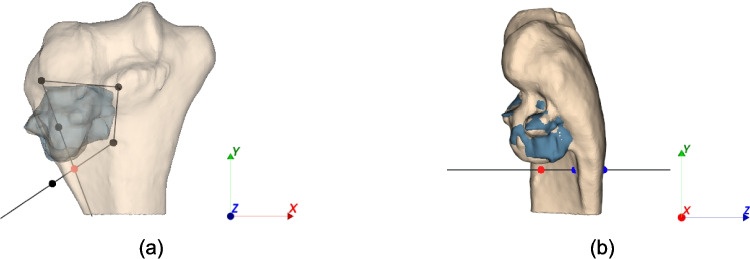
Procedure to handle self-intersecting surfaces: **a** polygonal chain generated by the n + 1 starting points and its point of intersection (red) and **b** ray projection of the polygonal chain intersection point that intersects bone mesh

Among the possible configurations explored by the PSO, it is possible that a self-intersecting cutting surface is generated. Generally, this does not represent a problem for clinical feasibility and indeed it is possible that a plausible solution is characterized by this situation, as shown in Fig. 6a. The intersection of two PSs consists of a segment; this segment can either be outside the bone and tumor surface, as in Fig. 6a, or it can cross the meshes as in Fig. 6b. When this latter situation is presented, the splitting between the cutting surface and the bone and tumor models computes a null result, causing the optimization process to fail. It is therefore necessary to detect such configurations and penalize them; evidently, such configurations are not valid from a clinical point of view since they do not ensure the resection of the tumor and do not guarantee that valid cutting guides can be created, and thus can be entirely avoided. To correctly discriminate such conditions, the following method was developed:

1. The polygonal chain generated by the $n+1$ points is considered, and its intersection point is found (Fig. 7a).
2. The ray projection in the $z$-direction of the intersection point is generated and it is checked whether this ray intersects the mesh of the bone or tumor (Fig. 7b).

The solutions identified as not valid were penalized in the optimization by assigning an arbitrarily high value to the objective function.

### Approximated convex hull solution for PSO initialization

**Fig. 8:**
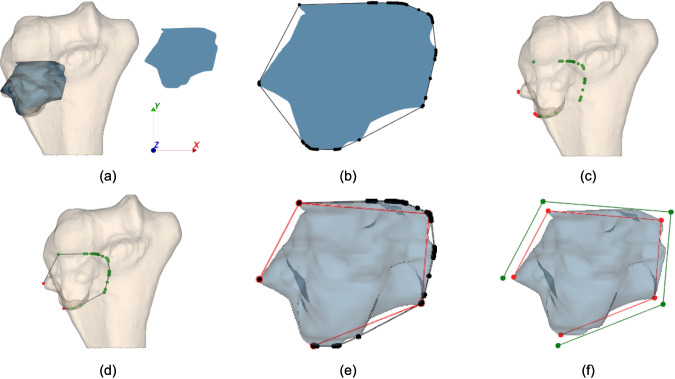
Algorithm to obtain a plausible solution to initialize PSO for a tumor case with *n* = 4: **a** tumor mesh projected on the *xy* plane; **b** convex hull of the tumor projected mesh: **c** true points (in green) and false points (in red); **d** polygonal chain of preserved points: all true points and two false points are preserved; **e** polygonal chain simplified (in red) through Ramer Douglas Peucker algorithm; and **f** in red the polygonal chain obtained through RDP, in green its corresponding offsetted polygonal chain

The approach for defining the cutting surface position introduces $2(n+1)$ variables, thus increasing the research space dimension with each additional cutting plane. This could lead to the necessity of greatly increasing the resources given to the algorithm in terms of number of iterations and number of particles in order to ensure the accurate exploration of the research space, causing, however, an increment in the time required for the optimization. Addressing this, the PSO is initialized with a plausible solution derived from an approximated convex hull (ACH) of the tumor mesh, such that the particle motion is directed towards a region close to the global minimum. The ACH solution is generated as follows and illustrated in Fig. 8 for a tumor case with $n = 4$:

1. Project the tumor mesh onto the *xy* plane (Fig. 8a).
2. Compute the 2D convex hull of the projection and order it by polar coordinates from the tumor centroid (Fig. 8b).
3. Generate $z$-projections for all points. Mark points whose $z$-projections intersect the bone as “true” (green in Fig. 8c) and the others as “false” (red in Fig. 8c).
4. Preserve all “true” points and the two “false” points that respectively precede and succeed a “true” point, since these are the only points that generate PSs intersecting the bone mesh. Generate a polygonal chain ordering the preserved points by setting the two preserved false points as first and last (Fig. 8d)
5. Use Ramer-Douglas-Peucker algorithm to simplify the preserved point set to $n+1$ points (Fig. 8e).
6. Apply a small offset to this point set to ensure no tumor intersection (Fig. 8f).

Although the ACH solution envelops the tumor, PSO refinement is still required to minimize healthy bone resection since offset applied in step 5 does not ensure that the PSs are tangent to the tumor mesh.

## Results and discussions

### Research of optimal optimization parameters

Particle swarm optimization involves hyperparameters $\omega$, ${c}_{1}$, ${c}_{2}$ that balance exploration of the research space and exploitation of the best solutions. Small $\omega$ allows convergence towards best solutions, while high ω encourages exploration around those solutions; ${c}_{1}$ defines the influence of personal best solutions and ${c}_{2}$ defines the influence of the global best solution.

The algorithm was tested with 9 constant $\omega$, ${c}_{1}$, ${c}_{2}$ configurations, setting $\omega =0.8$ and changing ${c}_{1}$ e ${c}_{2}$ satisfying ${c}_{1}+ {c}_{2} =4$ [16], and 1 configuration with $\omega$, ${c}_{1}$, ${c}_{2}$ varying through iterations to transition from exploration to exploitation.

**Fig. 9:**
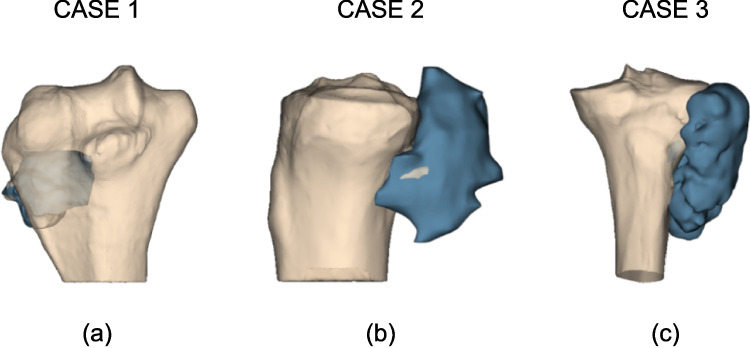
**a** Case n.1: tumor case localized near the distal humerus; 4 planes were used to execute resection; **b** Case n.2: tumor localized near proximal tibia; 2 planes were used to execute resection; and **c** Case n.3: tumor case localized near proximal tibia; 4 planes were used to execute resection

Three realistic bone tumor cases located in the epiphyses of long bones, specifically embedded in the humeral or tibial bone with a different number of planes ($n = 4, 2, 4$) were tested (Fig. 9). Bones were segmented from CT images with a pixel size of 0.3 mm and slice thickness of 0.6 mm while tumor 3D models from MRI images with a pixel size of 0.8 mm and slice thickness of 0.8 mm. Since MRI and CT were acquired using different devices that did not share the same reference system, MRI images were registered to CT images prior to the segmentation. The registration and segmentation procedures were carried out using the software Materialise Mimics 26 [17].

In all cases, a realistic surgical approach direction was selected with the help of an orthopedic surgeon. For each configuration, 10 runs on each case (300 total) were performed with 150 particles, 100 iterations, and random particles initialization within the bone bounding box limits. Tests were run on an AMD Ryzen 7 5700U processor with 16 GB RAM.

Table 1 shows mean, standard deviation, and confidence interval of the objective function for all 10 parameter configurations and for each of the three cases. Variable parameters $\omega$, ${c}_{1}$, ${c}_{2}$ proved to be the most effective configuration for Cases 1 and 3 ($n=4$), both in terms of mean and standard deviation, meaning respectively lower healthy bone removal and greater stability of the algorithm.

**Table 1:** Mean, standard deviation, and confidence interval of the objective function for all cases and parameter configurations

**CASES**	METRICS	Variable Coeff.	*c*_1_ = 4 *c*_2_ = 0	*c*_1_ = 3.5 *c*_2_ = 0.5	*c*_1_ = 3 *c*_2_ = 1	*c*_1_ = 2.5 *c*_2_ = 1.5	*c*_1_ = 2 *c*_2_ = 2	*c*_1_ = 1.5 *c*_2_ = 2.5	*c*_1_ = 1 *c*_2_ = 3	*c*_1_ = 3.5 *c*_2_ = 0.5	*c*_1_ = 0 *c*_2_ = 4
CASE 1	Mean	0.25757	0.39146	0.28186	0.27474	0.27835	0.27469	0.27569	0.29074	0.28612	0.31555
	Std Dev	0.00657	0.03986	0.01321	0.01171	0.00975	0.01089	0.01025	0.01529	0.01772	0.04068
	CI	0.00407	0.02470	0.00819	0.00726	0.00604	0.00675	0.00635	0.00947	0.01098	0.02521
CASE 2	Mean	0.11386	0.24666	0.12093	0.11069	0.11909	0.11915	0.11039	0.12155	0.13648	0.13636
	Std Dev	0.01281	0.06425	0.01632	0.00130	0.01670	0.01650	0.00096	0.01576	0.03564	0.02104
	CI	0.00794	0.03982	0.01011	0.00081	0.01035	0.01023	0.00060	0.00977	0.02209	0.01304
CASE 3	Mean	0.12109	0.31090	0.13801	0.13702	0.14093	0.14205	0.12334	0.16910	0.15034	0.17996
	Std Dev	0.00309	0.07596	0.01508	0.01559	0.02145	0.02420	0.00439	0.04620	0.02432	0.03679
	CI	0.00192	0.04708	0.00935	0.00966	0.01330	0.01500	0.00272	0.02863	0.01507	0.02280

For Case 2 ($n=2$), configurations ${c}_{1} = 1.5, {c}_{2} = 2.5$ and ${c}_{1} = 3, {c}_{2} = 1$ performed similarly to variable parameters. Fewer planes reduce research space complexity, allowing the global minimum to be found even with configurations biasing particles toward individuality (e.g., ${c}_{1} = 3.5$, ${c}_{2}= 0.5$) or the global best (e.g., ${c}_{1}= 0.5$, ${c}_{2}= 3.5$). Reducing algorithm resources (iterations and particles) for lower $n$ could decrease computation time while still ensuring to find the minimum. That being said, completely individualistic (${c}_{1}= 4$, ${c}_{2} = 0$) or globally-biased (${c}_{1}= 0$, ${c}_{2}= 4$) configurations performed poorly across all cases.

**Fig. 10:**
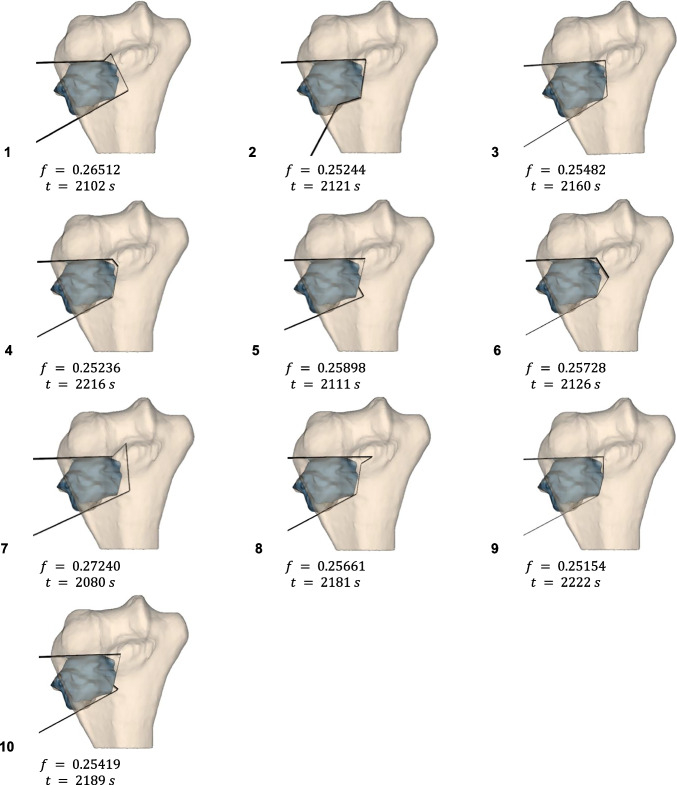
Cutting surfaces, their objective function values and optimization times, obtained with variable parameters for Case n.1

**Fig. 11:**
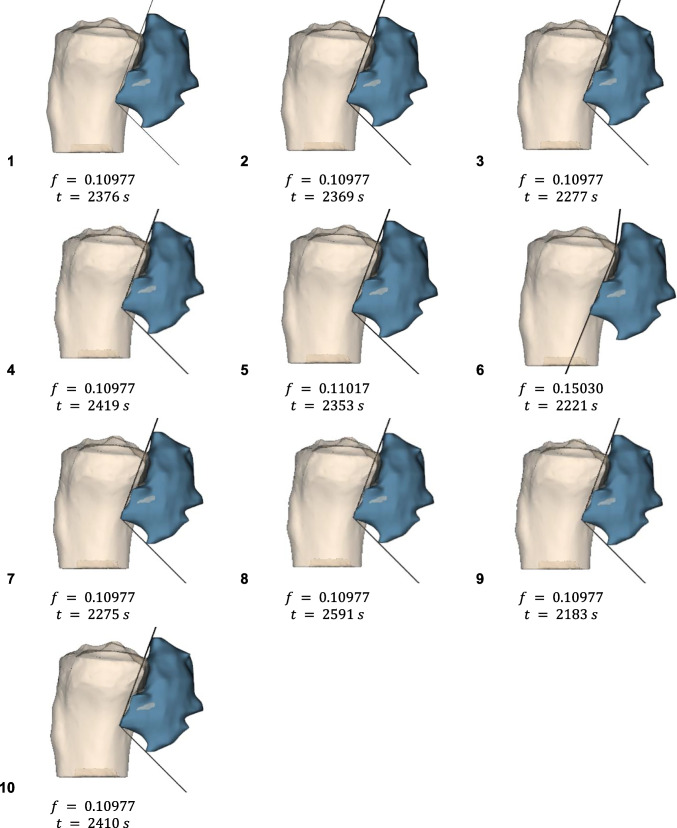
Cutting surfaces, their objective function values and optimization times, obtained with variable parameters for Case n.2

**Fig. 12:**
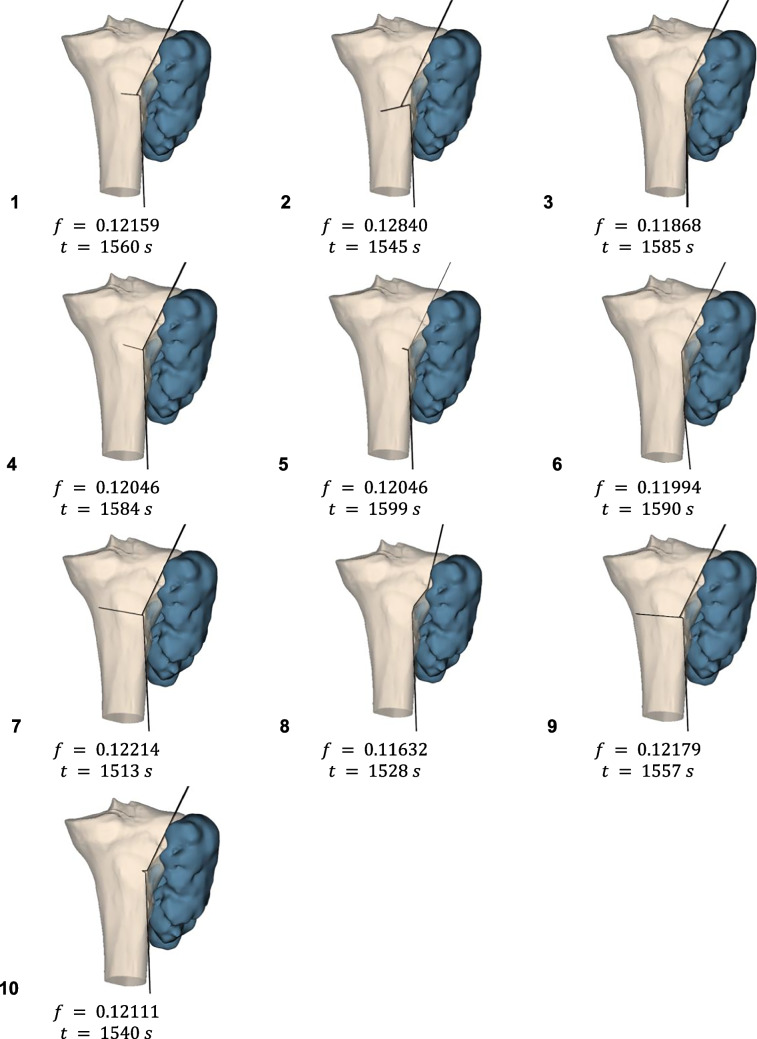
Cutting surfaces, their objective function values and optimization times, obtained with variable parameters for Case n.3

The final solutions achieved by the algorithm, using the selected configuration of parameters (i.e., variable coefficients) are depicted in Figs. 10, 11, and 12, together with their objective function values and optimization times for each test repetition.

For Case 2 ($n = 2$), highly similar solutions with nearly identical objective values were observed, likely indicating the lower number of variables allowed consistent identification of the global minimum solution.

For Cases 1 and 3 ($n=4$), while objective values were comparable, the cutting surface configurations differed, suggesting that higher $n$ increased difficulty in founding the global minimum. In all cases, it was observed that the objective function defined in the present study was a good descriptor for the problem faced, as the algorithm was guided to valid solutions.

In Figs. 10 and 12, it can be noted that some solutions have PSs with a very small length on the $xy$ plane. This has led to additional considerations, as such configurations might be a problem from a surgical point of view as it is necessary to guarantee the insertion of the saw, whose size is generally no less than 20 mm. It may therefore be appropriate to include such a constraint within future implementations of the algorithm. This constraint will surely entail a higher consumption of healthy bone but, at the same time, would guarantee the practical feasibility of the solution.

Case 3 often presented coincident redundant planes, indicating the selected $n=4$ may be excessive without much bone preservation benefit. To avoid the generation of such a surface, it might be effective to introduce a penalty in those solutions that present consecutive PSs that form small angles. This feature, however, needs to be carefully studied as it could hinder the identification of a good solution that is characterized by a lower number of planes; consequently, such constraint should be implemented only while allowing the algorithm also to explore solutions characterized by $n-1$ planes.

### Results with ACH solution initialization

Specific tests validated the ACH initialization method (Sect. 2.5) with 10 runs per tumor case using 100 iterations, variable coefficients, and 150 particles, one of which has been replaced with the ACH solution.

Table 2 compares the objective function mean, standard deviation, and confidence interval to previous runs with variable coefficients (Sect. 3.1). For Case 2 ($n = 2$), the same solution was found, confirming that the global minimum was reached for the smaller research space, but initialization was helpful in perfecting stability, as can be seen from the lower standard deviation.

**Table 2:** Mean, standard deviation, and confidence interval of the objective function for test with and without ACH initialization

**CASES**	METRICS	With ACH initialization	Without ACH initialization
CASE 1	Mean	0.24792	0.25757
	Std Dev	0.00062	0.00657
	CI	0.00038	0.00407
CASE 2	Mean	0.10977	0.11386
	Std Dev	1.46E-17	0.01281
	CI	9.07E-18	0.00794
CASE 3	Mean	0.11446	0.12109
	Std Dev	4.4E-05	0.00309
	CI	2.75E-05	0.00192

For Cases 1 and 3 ($n=4$), ACH initialization effectively reduced resected healthy bone (lower mean) and reached the same stable global minimum across all 10 runs (low standard deviation), unlike non-initialized tests. This is due to the fact that ACH initialization focuses particle exploration around a good initial solution, avoiding convergence to local minima.

**Fig. 13:**
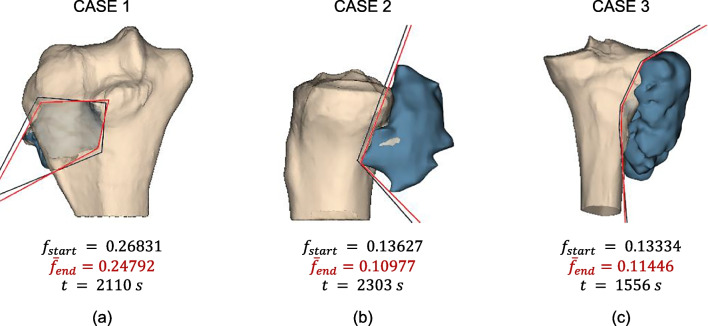
Cutting surface used for initialization (in black) and cutting surface after optimization (in red), their corresponding *f* values, and optimization time for **a** Case n.1, **b** Case n.2, and **c** Case n.3

Figure 13 shows the final cutting surfaces (red) compared to the initial ACH solutions (black), confirming optimization was necessary and effective in reducing healthy bone removal. Case 3 exhibited nearly coincident planes, reaffirming that using fewer planes (probably $n=3$) may suffice.

Figure 13 shows also optimization times. These are dependent on the number of points of bone and tumor 3D models and on the machine characteristics. Tests were performed on a common CPU with integrated GPU and still the global minimum was reached in about 1500–2200 s (about 25–35 min) which represents a time span widely compatible with the current planning phase of tumor resection surgeries. This time could easily be reduced by improving the machine capabilities.

Overall, ACH initialization proved valuable for guiding PSO toward the global minimum, especially for cases with a higher number of planes. Furthermore, it can be noted that PSO initialization could also be used to refine a solution provided by the surgeon, who might consider other aspects besides the minimization of resected bone.

## Conclusions and future work

This work implements an algorithm using particle swarm optimization to automatically position cutting planes, minimizing healthy bone removal while ensuring tumor resection. The method optimizes the cutting path generated by coincident planes following the tumor surface, even with concave geometries, unlike other literature approaches. The planes are parallel to a preferred surgical approach direction, reducing numerical complexity with minimal impact on effectiveness. Future work could use this 2D optimized path as a starting point for 3D plane optimization by allowing tilting relative to the *z*-direction. A procedure based on simplifying the tumor’s convex hull was devised to derive a plausible initial cutting surface configuration for initializing the PSO.

Preliminary tests on three tumor cases in long bone epiphyses, without initializing, effectively found the global minimum for cases with two planes but struggled with four planes due to the greater number of variables. Adapting resources for the optimization to the number of planes could improve stability.

Introducing constraints on plane angles and lengths could enhance solution feasibility. Using different variables to define plane positions could reduce the dimension of the research space. Additionally, in the future it is also planned to integrate the algorithm with a system for evaluating the optimal number of cutting planes, thus removing this parameter from the input data.

Tests with initialization showed significant improvements in resected healthy bone volume and solution stability, even for four planes. This approach could also be used to refine plane configurations provided by the surgeon who might consider other aspects beyond bone removal minimization.

The time required was not considered relevant since for each future case optimization needs to be performed a limited number of times. Furthermore, using higher-performance machines could easily reduce time resources.
